# The effectiveness of Korean medicine treatment in male patients with infertility: a study protocol for a prospective observational pilot study

**DOI:** 10.1097/MD.0000000000009696

**Published:** 2018-01-26

**Authors:** Kwan-II Kim, Junyoung Jo

**Affiliations:** a Department of Clinical Korean Medicine, College of Korean Medicine, Kyung Hee University; b Department of Korean Obstetrics and Gynecology, Conmaul Hospital of Korean Medicine, Seoul, Republic of Korea.

**Keywords:** asthenozoospermia, male infertility, oligozoospermia, semen analysis, sperm, traditional Korean medicine

## Abstract

Male factor subfertility has increasingly been considered the cause of infertility in couples. Many men with male infertility have sperm problems such as oligozoospermia, asthenozoospermia, or teratozoospermia. Because abnormal semen parameters are idiopathic to some extent, no standard therapy has been established to date. Herbal medicine has been reported to have beneficial properties in the treatment of subfertility, especially in improving semen quality both in vivo and in human studies. Therefore, we intend to investigate the effectiveness and safety of treatment using Korean medicine (KM) for infertile male patients with poor semen quality.

This will be a single-center, prospective, case-only observational pilot study. About 20 male patients with infertility who visit Conmaul Hospital of Korean Medicine will be recruited. We will follow the standard treatment protocol, which has shown good results in the treatment of male infertility. The protocol is composed mainly of a 10-week herbal decoction treatment; acupuncture and/or pharmacopuncture are added when needed. Semen samples, quality of life, and the scrotal temperatures of infertile men will be observed before and after the 10-week treatment with KM.

The study has received ethical approval from the Public Institutional Review Board (approval number: P01-201708-21-008). The findings will be disseminated to appropriate audiences via peer-reviewed publication and conference presentations.

Trial registration: Korean Clinical Trial Registry (CRIS), Republic of Korea: KCT0002611.

## 1 Introduction

Male factor subfertility accounts for at least half of all cases of subfertility,^[1]^ including oligozoospermia (low-spermatozoa count), asthenozoospermia (poor sperm motility), and teratozoospermia (abnormal sperm morphology).^[2]^ The cause of the abnormal semen parameters is idiopathic in 30% to 45% of men.^[2]^ Although some causes of male subfertility are treatable, treatment of idiopathic male factor subfertility remains empirical, with little evidence available.^[3]^ Based on limited evidence, antioxidant supplementation in subfertile men may improve live-birth rates for couples attending fertility clinics.^[4]^ Therefore, the use of assisted reproductive technology is usually the option of last resort in these situations. However, whether there are any differences in safety or effectiveness among the different treatments for male subfertility remains uncertain.^[5]^

Herbal medicine has been widely used to treat male infertility and has been shown to significantly improve both the quality and the quantity of sperm both in vivo^[6,7]^ and in human studies.^[8,9]^ Previously, we reported several studies of male idiopathic infertility in which the patients’ semen quality improved or the study participants were successful in fathering a baby spontaneously, despite poor semen quality, using Korean medicine (KM).^[10–12]^

In the present study, the effects of KM on the semen parameters of male infertility patients will be investigated prospectively to verify the clinical effects of KM therapies on male patients with poor semen quality.

## 2 Methods and design

### 2.1 Objective

To investigate the effectiveness of KM treatment in male infertility, we will evaluate whether KM treatment improves the sperm parameters of men suffering from infertility.

### 2.2 Design

**Figure 1:**
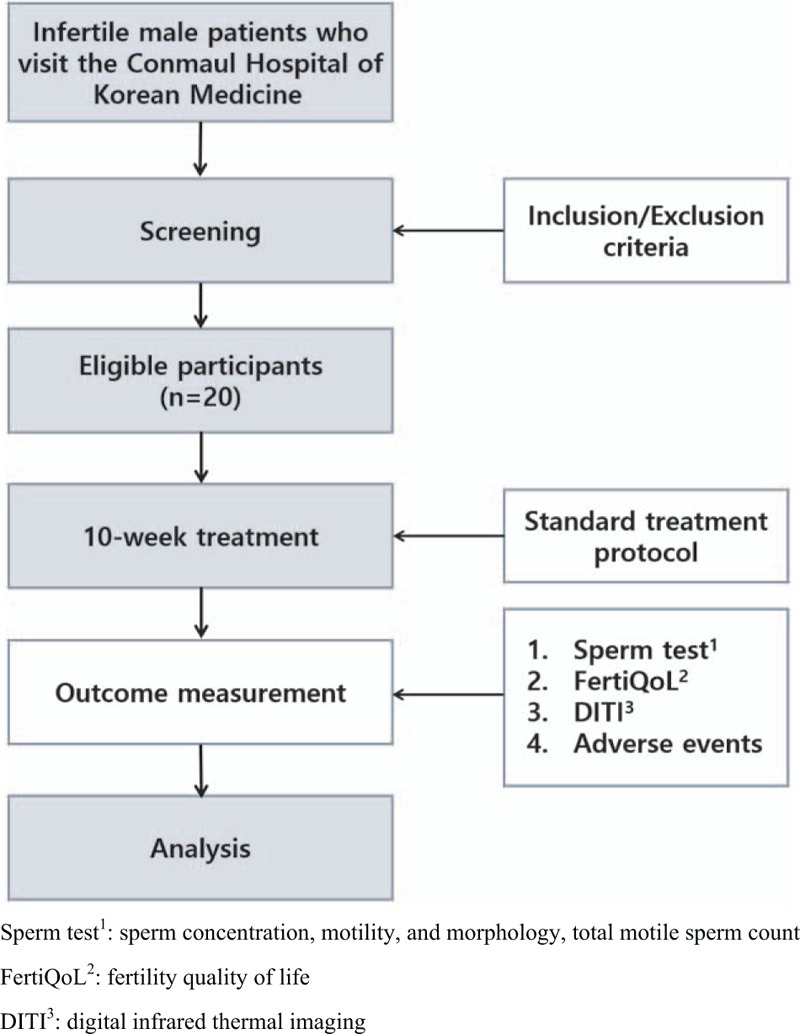
Flow chart of the study.

The study will be a single-center, prospective, case-only observational pilot study. Fig. 1 shows a flow chart of this study.

### 2.3 Ethics

The study has been authorized by the Public Institutional Review Board (approval number: P01-201708-21-008). The protocol accords with the Declaration of Helsinki. Signed informed consent forms will be obtained from all eligible participants before enrollment. This study is registered with the Korean Clinical Trial Registry, Republic of Korea: KCT0002611.

### 2.4 Participants

#### 2.4.1 Inclusion criteria

(1) Infertile men between 18 and 45 years of age;
(2) Men with infertility problems for a period of >1 year with poor semen quality. Poor semen quality will be defined by abnormal semen parameters observed within 2 weeks of visiting our hospital, including oligozoospermia, asthenozoospermia, teratozoospermia, or a combination of these 3 parameters, based on the 2010 World Health Organization (WHO) guidelines^[13]^;
(3) Study participants who provide written informed consent;
(4) Patients who trust the researchers, are willing to cooperate throughout the study, and comply with the study regulations.

#### 2.4.2 Exclusion criteria

(1) Patients with azoospermia;
(2) Patients taking antiserotonin, antidepressant, or other psychiatric drugs.

#### 2.4.3 Rejection and withdrawal criteria

(1) Voluntary withdrawal from the study;
(2) Participants who do not comply with the prescribed 10-week herbal decoction regimen (<80% of the prescribed doses);
(3) Patients not available for follow-up.

### 2.5 Recruitment

In total, 20 male patients with infertility will be recruited at the Conmaul Hospital of Korean Medicine. The investigator (JJ) will explain the objective of this study and the details of the procedures. After obtaining patient consent, screening will be carried out to assess study suitability. Participants will be free to withdraw at any time during the study, and this will not affect their clinical treatment.

### 2.6 Intervention

The participants will be treated with the standard protocol practiced at the Conmaul Hospital of Korean Medicine by JJ. The standard protocol for treatment of male infertility consists mainly of treatment with herbal medicine, with electronic acupuncture and/or pharmacopuncture added as needed. Herbal medicine is decocted daily from 14 herbs: Cuscutae Semen 12 g, Rehmanniae Radix Preparata 12 g, Epimedii Herba 12 g, Morindae Radix 10 g, Cynomorii Herba 10 g, Corni Fructus 10 g, Lycii Fructus 10 g, Dioscoreae Rhizoma 10 g, Rubi Fructus 10 g, Ginseng Radix 6 g, Cinnamomi Cortex 6 g, Poria Sclerotium 6 g, Plantaginis Semen 4 g, Schisandrae Fructus 4 g (Table 1). Study participants will take this herbal medicine, 120 mL twice a day for 10 weeks. If necessary, acupuncture with electrical stimulation or pharmacopuncture will be conducted once a week. The acupoints will be as follows: CV4, CV6, ST29, SP6, KI3, LR3, BL32, and BL23.^[14,15]^Table 2 shows details of the study procedure.

**Table 1:**
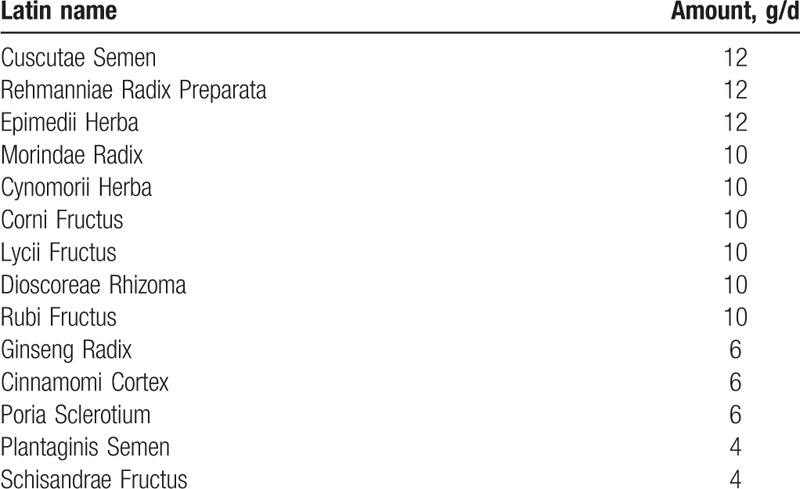
Components of Korean herbal medicine.

**Table 2:**
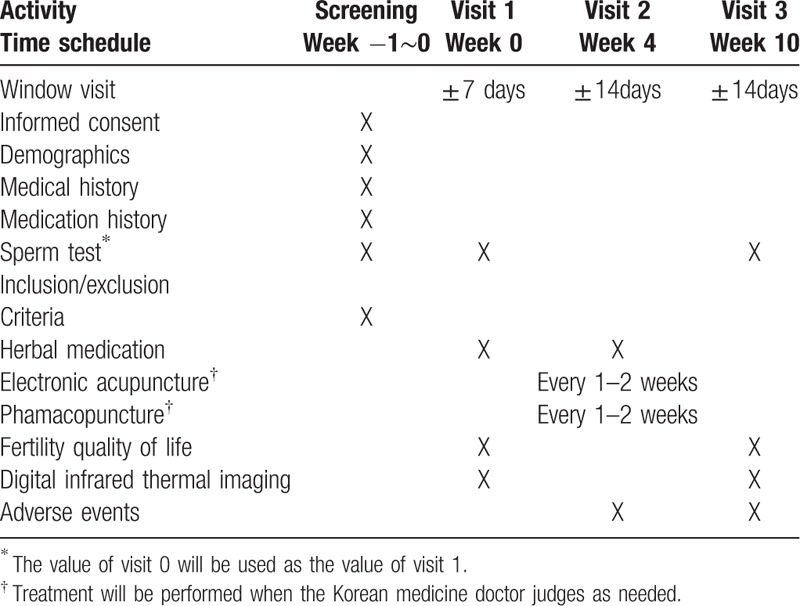
Data collection schedule.

### 2.7 Outcome measures

#### 2.7.1 Primary outcome

The primary outcomes assessed will be the mean difference in total motile sperm count and the values from each sperm test (sperm concentration, motility, and morphology [percentage normal in form]) from baseline to the end of treatment. Semen samples of men with infertility will be evaluated before and after the 10-week treatment with KM. The sperm test will be performed by experienced clinicians from the same hospital to reduce bias in the semen analysis.

#### 2.7.2 Secondary outcomes

The secondary outcomes assessed will be changes in the quality of life questionnaire and in digital infrared thermal imaging (DITI).

##### 2.7.2.1 Quality of life

The fertility quality of life (FertiQoL) tool is an international instrument that measures quality of life in infertile patients.^[16]^ FertiQoL has been validated in several languages, including Korean. FertiQoL consists of 36 items that assess overall life and physical health (2 items), along with core-related (24 items) and treatment-related (10 items) quality of life. Core subscales consist of emotional, mind–body, relational, and social domains. Treatment subscales comprise environment and treatment tolerability domains. FertiQoL is scored on a 5-point Likert scale (0–4). The sum score is converted to a 100-point scale, and higher scores indicate a better quality of life.

##### 2.7.2.2 DITI

Scrotal skin temperature affects sperm output. Scrotal hyperthermia results in impairment of spermatogenesis.^[17,18]^ DITI may provide reliable measurements of testes temperature.^[19]^ We will use DITI to evaluate scrotal skin temperatures from baseline to visit 3 to confirm the effects of KM on scrotal temperature.

### 2.8 Adverse events (AEs)

An adverse event (AE) is defined as an undesirable, unintended sign, symptom, or disease that does not necessarily have a cause–effect relationship with the intervention evaluated in the study. We will perform continuous monitoring of AEs and make any relevant decision in this regard on the basis of both objective and subjective signs.

### 2.9 Sample size

A pilot study for planning a larger study and estimating its effective size requires an adequate small sample size. The general rule is that at least 10^[20]^ or 12 participants^[21]^ per treatment group is adequate as the required sample size in a pilot study. This study should therefore include at least 12 participants; assuming a dropout rate of 40%, we have calculated the necessary initial sample size to be 20 participants.

### 2.10 Statistical analyses

We will analyze the results of treatment through comparisons of paired data because this will be a case-only series. Descriptive statistics will be used for continuous variables, and frequencies for categorical variables. Continuous variables will be analyzed using the paired *t*-test when the assumption of normality is satisfied; otherwise, the Wilcoxon signed-rank test will be used. Categorical variables will be analyzed using McNemar's test. All data will be analyzed using SPSS for Windows software. The level of statistical significance will be set at a 2-sided *P*-value of .05.

## 3 Discussion

Recently, a published meta-regression analysis showed a significant decline in sperm counts between 1973 and 2011, driven by a 50% to 60% decline among men from North America, Europe, Australia, and New Zealand who were not selected based on fertility.^[22]^ These findings strongly imply a significant decline in male reproductive health, which has serious implications beyond fertility concerns.^[22]^ A similar decline has been found in young Chinese men, whose semen parameters showed a decreasing trend over a 15-year observational period.^[23]^

Prescription of Korean herbal medicine for the treatment of oligozoospermia, asthenozoospermia, and/or teratozoospermia is common in clinics in Korea. However, only a few clinical case reports have evaluated the effectiveness and safety of KM.^[10–12]^ Therefore, more data are required to provide optimal information to infertile male patients with poor semen quality.

The herbal medicine used in this study has been used for several years to treat male reproductive diseases in Korea, including male infertility and sexual dysfunction. The composition of the medicine is similar to the medicine used in several studies that showed therapeutic effects for male infertility.^[7,8,12]^

Corni Fructus, Schisandrae Fructus, Rubi Fructus, Cuscutae Semen, and Lycii Fructus are herbs that are frequently used in KM to treat male infertility. Each of these 5 herbs exhibits antioxidant properties, and combinations of these herbs have been shown to improve both sperm count and sperm activity.^[6]^ In traditional Chinese medicine, Morindae Radix is widely used to invigorate the kidneys, to support yang (the original energy in the human body) in resisting diseases, and to treat male infertility.^[24]^ An in vitro study showed that oligosaccharides from Morindae Radix acted as a protective agent against oxidative damage to sperm DNA.^[25]^ A mixture of 2 herbal extracts consisting of Epimedii Herba and Angelicae Gigantis Radix increased sperm production by reducing oxidative stress and had a positive effect in a male infertility model.^[26]^ Cynomorii Herba was associated with significant increases in epididymal sperm counts and absolute testes weights in rat testes.^[27]^ These effects might be because of these herbs’ antioxidative properties, which help to restore imbalances caused by excessive levels of reactive oxygen species.

This observational study will allow collection of valuable and reliable data to evaluate the effectiveness of a specific KM protocol for treating male infertility. This study will contribute to the clinical evidence regarding the effectiveness of KM for infertile men with poor semen quality.
